# Fertility-sparing re-treatment for endometrial cancer and atypical endometrial hyperplasia patients with progestin-resistance: a retrospective analysis of 61 cases

**DOI:** 10.1186/s12957-024-03439-w

**Published:** 2024-06-25

**Authors:** Junyu Chen, Dongyan Cao

**Affiliations:** 1 Department of Obstetrics and Gynecology, Qilu Hospital of Shandong University, Jinan, 250012 China; 2 Department of Obstetrics and Gynecology, Peking Union Medical College Hospital, National Clinical Research Center for Obstetric & Gynecologic Diseases, Chinese Academy of Medical Sciences & Peking Union Medical College, Beijing, 100730 China

**Keywords:** Endometrial cancer, Atypical endometrial hyperplasia, Fertility-sparing re-treatment, Progestin resistant

## Abstract

**Objective:**

This study aimed to evaluate the oncological and reproductive outcomes of fertility-preserving re-treatment in progestin-resistant endometrial carcinoma (EC) and atypical endometrial hyperplasia (AEH) women who desire to maintain their fertility.

**Methods:**

Our study included 61 progestin-resistant EC/AEH patients. These patients underwent treatment with gonadotropin-releasing hormone agonist (GnRHa) solely or a combination of GnRHa with levonorgestrel-releasing intrauterine system (LNG-IUD) or aromatase inhibitor (AI). Histological evaluations were performed every 3–4 months. Upon achieving complete remission (CR), we recommended maintenance treatments including LNG-IUD, cyclical oral contraceptives, or low-dose cyclic progestin until they began attempting conception. Regular follow-up was conducted for all patients. The chi-square method was utilized to compare oncological and fertility outcomes, while the Cox proportional hazards regression analysis helped identify risk factors for CR, recurrence, and pregnancy.

**Results:**

Overall, 55 (90.2%) patients achieved CR, including 90.9% of AEH patients and 89.7% of EC patients. The median re-treatment time was 6 months (ranging from 3 to 12 months). The CR rate for GnRHa alone, GnRHa + LNG-IUD and GnRHa + AI were 80.0%, 91.7% and 93.3%, respectively. After a median follow-up period of 36 months (ranging from 3 to 96 months), 19 women (34.5%) experienced recurrence, 40.0% in AEH and 31.4% in EC patients, with the median recurrence time of 23 months (ranging from 6 to 77 months). Among the patients who achieved CR, 39 expressed a desire to conceive, 20 (51.3%) became pregnant, 11 (28.2%) had successfully deliveries, 1 (5.1%) was still pregnant, while 8 (20.5%) suffered miscarriages.

**Conclusion:**

GnRHa-based fertility-sparing treatment exhibited promising oncological and reproductive outcomes for progestin-resistant patients. Future larger multi-institutional studies are necessary to confirm these findings.

## Introduction

Endometrial cancer (EC) is one of the most prevalent and increasingly challenging gynecological malignancies, with its incidence continually rising in recent years [1–3]. Approximately 15% of EC cases are observed in premenopausal women, and 5% in women of child-bearing age [4]. Thus, conservative treatments are implemented in young patients with early-stage EC or AEH who wish to preserve their fertility.

High-dose progestin is the mainstay of conservative treatments for AEH and EC, with medroxyprogesterone acetate (MPA) and megestrol acetate (MA) being the most frequently used drugs [5]. The Complete remission (CR) rate for progestin therapy is around 70%, however, about 30% of patients show insensitivity to progestin therapy, a condition termed as progestin-resistance [6]. So far, the precise mechanisms underlying progestin-resistance remain elusive. These patients often give up fertility preservation and undergo hysterectomy with bilateral salpingo-oophorectomy, potentially including the resection of sentinel lymph nodes or pelvic or para-aortic lymphadenectomy, resulting in permanent loss of fertility. Nevertheless, some patients still have a strong desire for childbearing. Some studies have suggested that fertility-sparing re-treatment is a viable option for progestin-resistant patients [7]. Herein, identifying an alternative treatment for such patients and evaluating its efficacy and safety is crucial.

Currently, gonadotropin-releasing hormone agonist (GnRHa) is considered as a potential alternative to oral-systemic progestin for treating women with EC and AEH [8]. Our previous work revealed that the combination of GnRHa with levonorgestrel-releasing intrauterine system (LNG-IUD) or aromatase inhibitors (AI) was a promising option for preserving fertility in women diagnosed with EC/AEH, exhibiting a favorable response [9–11]. However, the existing evidence on effective application of GnRHa in progestin-resistant patients is limited, necessitating further research.

This study aimed to assess the efficacy and safety of fertility-sparing re-treatment in progestin-resistant women diagnosed with EC or AEH. Our findings could provide a more comprehensive reference for fertility-sparing treatment in EC.

## Method

### Patients recruited

Progestin-resistance was defined as the persistence or progression of the disease after more than 6 months of regular oral high-efficiency progestin treatment (MPA, 500 mg/d, or MA, 320 mg/d) [12, 13]. Including no or partial pathological change for the disease or AEH progresses to EC. Patients were recruited from the Department of Obstetrics and Gynecology at Peking Union Medical College Hospital (PUMCH) and Qilu hospital of Shandong University between January 2011 and June 2022.

All patients were fully informed and re-evaluated, still meeting the criteria for fertility preservation therapy. The inclusion criteria were as follows: [1] women aged 18–40 years desiring to preserve their fertility; [2] histologically confirmed AEH or EC, grade 1; [3] no signs of myometrial invasion or extra-uterine metastasis confirmed by magnetic resonance imaging; [4] patients with progestin-resistant; [5] written informed consent from the patient; [6] regular followed-ups with complete data available.

### Treatment methods

1. GnRHa: subcutaneous injection of 3.75 mg GnRHa every 4 weeks;
2. GnRHa + LNG-IUD: a combination of subcutaneous injection of 3.75 mg GnRHa every 4 weeks and continuous Mirena insertion;
3. GnRHa + AI: combination of subcutaneous injection of 3.75 mg GnRHa every 4 weeks and daily oral administration of 2.5 mg Letrozole.

The choice of these three regimes was based on the physicians’ recommendation and patients’ preference. All patients received health education during treatment and were screened for metabolic diseases such as hyperglycemia and hyperlipidemia. Weight and body fat were also measure. Weight loss was achieved through diet control combined with physical exercise, and blood sugar was managed accordingly in patients diagnosed with diabetes. A multidisciplinary management involving the departments of nutrition, endocrinology, and reproduction was employed.

During treatment, outpatient visits were arranged, symptoms such as vaginal spotting and abdominal pain were recorded, and physical examinations, including body weight, complete blood counts, and biochemistry panels were performed. Transvaginal ultrasound was conducted at every visit to assess the endometrium. Histological response was determined by endometrial biopsy under hysteroscopic evaluation every 3–4 months (one course) during treatment.

### Response evaluation

Pathological responses to treatment were categorized as complete response (CR), partial response (PR), stable disease (SD), and progressive disease (PD). CR was defined as the absence of evidence of hyperplasia or carcinoma. PR as regression of AEH or EC to hyperplasia without atypia. SD as the persistence of disease as initially diagnosed, and PD as progression to a higher grade lesion, including myometrial invasion, extra-uterine disease, or lymph node metastasis [14]. Patients with a PR or SD continued treatment for an additional 1–2 courses, while those with PD were immediately recommended to undergo hysterectomy. Those who did not achieve CR after 6 months of therapy were considered to have failed fertility-preserving treatment and were recommended for surgery. Once CR was achieved, patients who desired pregnancy were encouraged to conceive or referred for assisted reproductive technology (ART). Patients in CR without plans for childbirth soon were prescribed maintenance therapy including oral contraceptives, cyclic progestin, or LNG-IUD insertion to prevent recurrence.

### Follow-up

After achieving CR, all patients were regularly followed up at intervals of 3–6-month. During each visit, data relating to menstrual period or abnormal vaginal bleeding, results of trans-vaginal ultrasound scan or magnetic resonance imaging if necessary, and information about relapse and pregnancy was documented. If a patient underwent hysterectomy, the reason for and the histological results of the surgery were also collected.

### Statistical analysis

Statistical analysis was performed using IBM SPSS Statistics for Windows (version 22.0; IBM Corp Armonk, NY). Categorical variables are summarized in frequency tables, while continuous variables such as Body mass index (msystem), time of CR, recurrence, and follow-up, were presented as median (range: min–max). Frequency distributions were compared using Chi-square tests, and median values were compared using Mann-Whitney U test. Cox regression models were constructed to determine the associations between factors and CR, recurrence, and fertility. Differences were considered statistically significant at *P* < 0.05.

## Results

### Characteristics of progestin-resistant patients

A total of 61 patients were included in this study, all of whom were seeking to preserve reproductive function following the failure of oral progestin therapy and who still met the criteria for fertility-sparing therapy. Among them, 14 (23.0%) patients were diagnosed with AEH, and 47 (77.0%) were diagnosed with EC before treatment. The median BMI was 26.43 kg/m^2^ (18.87–41.49 kg/m^2^), with 14 (23.0%) patients being overweight (BMI:24–28 kg/m^2^) and 27 (44.3%) patients being obese (BMI ≥ 28 kg/m^2^). Eighteen (29.5%) women had comorbidities of polycystic ovary syndrome (PCOS), 6 (9.8%) had DM, and 5 (8.2%) patients had hypertension. At the initial of treatment, 35(57.4%) patients were treated with MPA at a dose of 500 mg/d, and 26 (42.6%) patients with MA at a dose of 320 mg/d.

After a median of 6 months of progestin treatment (range: 3–12 months), 11 (18.0%) EC patients achieved PR (reversed to AEH but remained stable in AEH for at least 6 months), 47 (77.1%) patients showed SD (11 in AEH and 36 in EC), and 3 (4.9%) AEH patients progressed to EC. Thus, before re-treatment, 22 (36.1%) patients were diagnosed with AEH and 39 (63.9%) were diagnosed with EC. The median age at this time was 32 years (range: 22–40 years). Regarding the choice of regimens, 10 patients received the GnRHa regimen alone, 15 patients received the GnRHa + AIs regimen and 36 patients received the GnRHa + LNG-IUD regimen. The clinical characteristics of patients are summarized in Table 1.

**Table 1:** Clinicopathologic characteristics of the patients

Characteristics	Values, *n*(%)
N	61
Age, years (median, range)	32 (22–40)
BMI, kg/m^2^ (median, range)	26.43 (18.87–41.49)
Nulliparous	42 (68.9%)
Comorbidity	
PCOS	18 (29.5%)
DM	6 (9.8%)
HP	5 (8.2%)
Obesity	27 (44.3%)
Histology (before progestin treatment)	
AEH	14 (23.0%)
EC	47 (77.0%)
Progestin regimen	
MPA	35 (57.4%)
MA	26 (42.6%)
Progestin time, months (median, range)	6 (3–12)
Treatment outcome	
PR	11 (18.0%)
SD	47 (77.1%)
PD	3 (4.9%)
Histology (After progestin treatment)	
AEH	22 (36.1%)
EC	39 (63.9%)
Regimens for re-treatment	
GnRHa	10 (16.4%)
GnRHa + Letrozole	15 (24.6%)
GnRHa + Mirena	36 (59.1%)

Notes: BMI, Body mass index; PCOS, Polycystic ovary syndrome; DM, Diabetes mellitus; HP, Hypertension; AEH, Atypical endometrial hyperplasia; EC, Endometrial carcinoma; MPA, Medroxyprogestin; MA, Megestrol acetate; PR, Partial response SD, Stable disease; PD, Progressive disease; GnRHa, Gonadotrophin releasing hormone agonist

### Treatment outcome

**Fig. 1:**
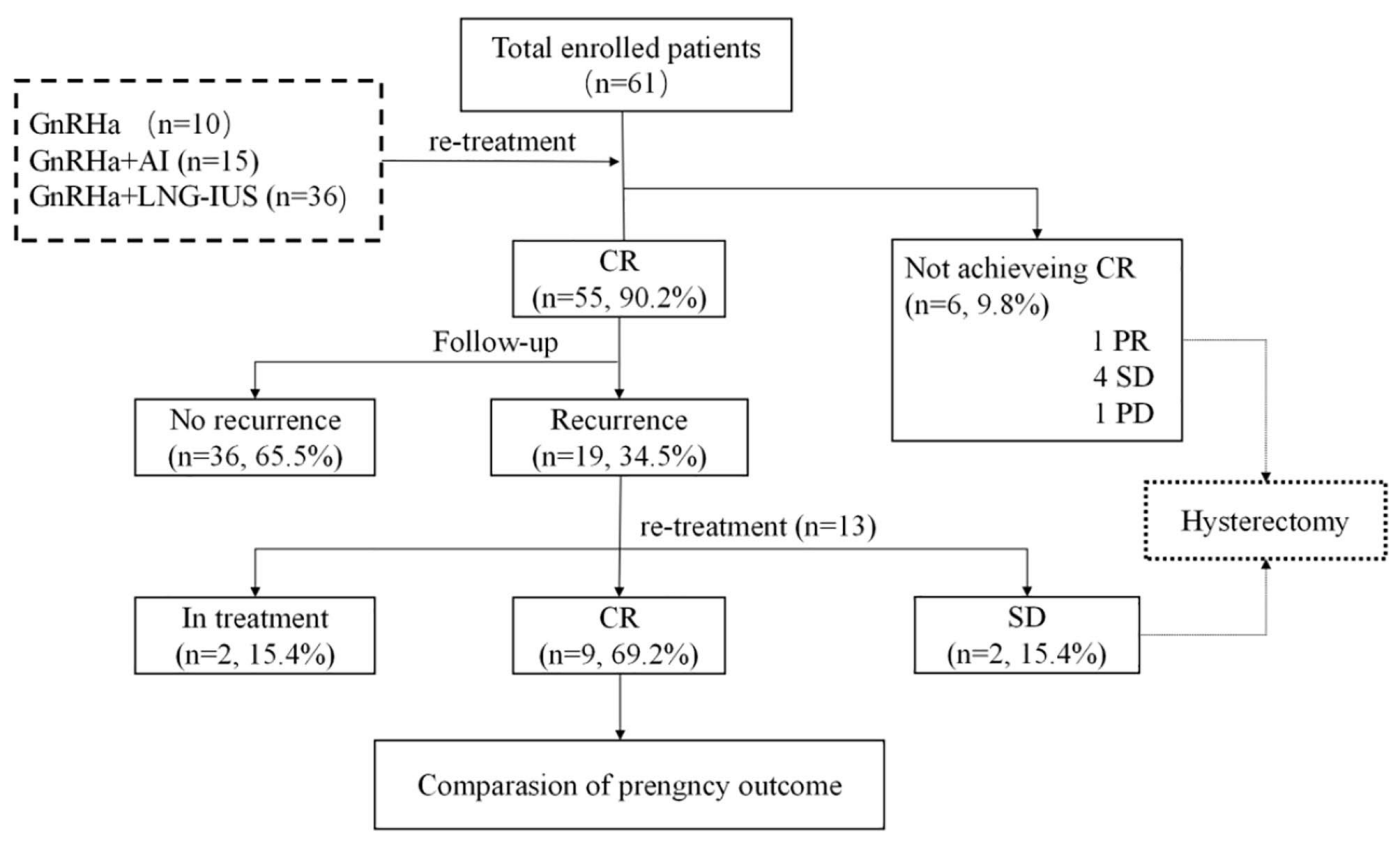
The flow of the study

In total, 55 (90.2%) patients achieved CR with a median re-treatment time of 6 months (range: 3–12 months) (Table 2). There were 6 (9.8%) patients who failed to achieve CR, which included 1 patient with PR, 4 with SD and, 1 with PD. These patients subsequently underwent hysterectomy with or without lymphadenectomy (Fig. 1). According to the postoperative pathological diagnosis, of these 6 patients who failed re-treatment, one was diagnosed with AEH, five were diagnosed with stage IA endometrioid adenocarcinoma. Among the stage IA EC patients, 3 patients with lesions confined in endometrium, 2 patients with superficial myometrial infiltration. In addition, one patient was combined with stage IA1 cervical squamous cancer. All patients were alive without tumors at the final contact after a median follow-up time of 36 months (range: 3–96 months).

The CR rate was 90.9% in patients with AEH and 89.7% in patients with EC, with a median time of GnRHa administration being 5 months (range: 3–12 months) in patients with AEH, and 6 months (range: 3–12 months) in patients with EC (Table 2). The median total treatment duration for EC and AEH patients was 12 months (range: 6–21 months) and 9 months (range: 6–19 months), respectively.

**Table 2:** Oncological and Fertility outcomes of patients

Characteristics	Endometrial carcinoma (*n* = 39)	Atypical endometrial hyperplasia (*n* = 22)	Total (*n* = 61)
Complete remission			
Complete remission rate	35 (89.7%)	20 (90.9%)	55 (90.2%)
Re-treatment time, month (range)	6 (3-12)	5 (3-9)	6 (3-12)
Total time, month (range)	12 (6-21)	9 (6-19)	6 (3-12)
Follow-up			
Maintenance therapy	31 (88.6%)	16 (80%)	47 (85.5%)
Follow-up time, month (range)	30 (3–96)	42 (14–88)	36 (3–96)
Recurrence			
Recurrence rate	11 (31.4%)	8 (40.0%)	19 (34.5%)
Recurrence time, month (range)	15 (9–48)	28 (6–77)	23 (6–77)
Attempts to conceive	26	13	39
Live baby delivery	7 (26.9%)	4 (30.8%)	11 (28.2%)
In pregnancy	1 (3.8%)	0	1 (3.6%)
Miscarriage	6 (23.1%)	2 (15.4%)	8 (20.5%)
Total pregnancy rate	14 (53.8%)	6 (46.2%)	20 (51.3%)

After 3 months of treatment, the rate of CR was 40.9% in the AEH and 41.0% in the EC when endometrial pathology was obtained by the first hysteroscopy to evaluate efficacy. The second assessment after 6 months of treatment, showed a higher CR rate in AEH than in EC (77.3% vs. 66.7%). By the end of the third therapy course, over 80% of patients in both groups achieved CR (Table 3).

**Table 3:** Duration of complete remission

Times	AEH	EC	Total	*P*-value
3-months CR rate	40.9% (9)	41.0% (16)	41.0% (25)	0.893
6-months CR rate	77.3% (17)	66.7% (26)	70.5% (43)	0.373
9-months CR rate	90.9% (20)	82.1% (32)	85.2% (52)	0.435
Total	90.9% (20)	89.7% (35)	90.2% (55)	0.883

Notes: AEH = atypical endometrial hyperplasia, EC = endometrial carcinoma

CR rates in obese and non-obese patients were 97.1% and 81.5%, respectively (*P* = 0.042). High remission rates were observed in patients younger than 35 years old (90.9% vs. 81.5%, *P* = 0.753), in patients without PCOS (93% vs. 83.3%, *P* = 0.246), and in those who lost more than 10% of their initial weight (95.5% vs. 87.2%, *P* = 0.297), despite no statistical significance. The CR rate in GnRHa, GnRHa + AI and GnRHa + LNG-IUD was 80%, 93.3% and 91.7%, respectively. GnRHa combined therapy achieve a higher CR rate than GnRHa alone (92.2% vs. 80%, *P* = 0.238), with patients who received the GnRHa + AI regimen showing the highest CR rate (Table 4).

**Table 4:** Predictors of complete response

Predictors of complete response	Univariate analysis HR (95% CI)	*P*-value	Multivariate analysis HR (95% CI)	*P*-value	Ratio
Age (years): <35 vs. ≥35	1.222 (0.364–4.103)	0.753			90.9% vs. 88.2%
Obesity: no vs. yes	2.083 (1.286–3.375)	**0.042**	2.006 (1.149–3.049)	0.081	97.1% vs. 81.5%
AEH vs. EC	1.048 (0.575–1.909)	0.883			90.9% vs. 89.7%
Loss-weight:<10% vs. ≥ 10%	0.437 (0.071–2.695)	0.297			87.2% vs. 95.5%
PCOS: No vs. Yes	1.833 (0.738–4.511)	0.246			93.0% vs. 83.3%
Regimen: GnRHa vs. GnRHa combination	0.780 (0.438–1.388)	0.238			80.0% vs. 92.2%

Notes: BMI, Body mass index; PCOS, Polycystic ovary syndrome; AEH, Atypical endometrial hyperplasia; EC, Endometrial carcinoma; GnRHa, Gonadotrophin releasing hormone agonist; AI, Aromatase inhibitor; LNG-IUD, Levonorgestrel-releasing intrauterine system

### Adverse effects

Postmenopausal symptoms such as hot flashes and vaginal dryness were the most common adverse effects (16.4%), followed by irregular bleeding (14.8%). The degree of menopausal symptoms was mild, and no patients required add-back estrogen. One patient experienced IUD dislocation, which was resolved by reinserting the IUD. There were no recorded instances of weight gain, liver dysfunction, or thromboembolism were recorded. The scheduled treatment was not delayed due to these minor side effects, and no treatment-related deaths were identified.

### Maintenance therapy

After achieving pathological CR, 47 (85.5%) patients received maintenance treatment including LNG-IUD, cyclical oral contraceptives, and low-dose cyclic progestin, until they began attempts at gestation. The remaining 8 (14.5%) patients did not receive maintenance therapy and underwent regular follow-ups.

### Follow-up and recurrence

After a median follow-up period of 36 months (range: 3–96 months), 19 (34.5%) women experienced recurrence (Table 2). The median time to recurrence was 23 months (range: 6–77 months). After recurrence occurred, 6 patients underwent hysterectomy. Thirteen patients received fertility-sparing re-treatment after recurrence, with 7 being diagnosed as EC and 6 as AEH. Three patients received progestin therapy, 3 patients received GnRHa solely, and 7 patients received GnRHa + LNG-IUD, of whom 9 (69.2%) achieved CR again. Hysterectomy was performed on 2 (15.4%) patients due to SD, and both were diagnosed as stage IA EC according to postoperative histology. The remaining 2 patients were still in treatment at the final contact. One patient experienced a second recurrence and received the third-time fertility-sparing therapy, also achieved CR. No patients died of the disease during this period.

**Table 5:** Factors related to recurrence

Factors	Univariate analysis HR (95% CI)	*P*-value	Multivariate analysis HR (95% CI)	*P*-value	Ratio
Age (years): <35 vs. ≥35	0.853 (0.565–1.287)	0.464			28.6% vs. 38.2%
AEH vs. EC	1.152 (0.736–1.802)	0.520			40.0% vs. 31.4%
Obesity: No vs. Yes	0.924 (0.474–1.801)	0.817			33.3% vs. 36.4%
Maintenance therapy: No vs. Yes	1.380 (1.007–1.893)	**0.009**	3.512 (1.372–6.207)	**0.023**	75.0% vs. 27.6%
Loss-weight:<10% vs. ≥ 10%	2.243 (0.879–5.723)	0.057			44.1% vs. 19.0%
PCOS: No vs. Yes	0.462 (0.198–1.079)	0.073			27.5% vs. 53.3%
Regimen: GnRHa vs. GnRHa combination	1.023 (0.809–1.293)	0.849			37.5% vs. 34.0%
Conceive: No vs. Yes	1.629 (0.700-3.788)	0.229			41.2% vs. 25%

Notes: PCOS, Polycystic ovary syndrome; AEH, Atypical endometrial hyperplasia; EC, Endometrial carcinoma; GnRHa, Gonadotrophin releasing hormone agonist

Factors related to recurrence are shown in Table 5. Both univariate and multivariate analysis indicated that patients who receive maintenance therapy were at a lower risk for recurrence (75% vs. 27.6%, *P*<0.05). For the 8 patients who did not receive maintenance therapy, 6 (75%) of them experienced recurrence with a median recurrence time of 24.5 months (range: 12–36) months. Of the 6 patients who had recurrence, 4 opted for hysterectomy, and 2 underwent fertility-sparing re-treatment, with both achieving CR again. The 2 patients who opted for hysterectomy were diagnosed with stage IA EC on postoperative pathological examination. The remaining 2 patients who did not experience recurrence during the follow-up period continued to be disease-free at their last follow-up visit.

The recurrence rate was 40% in AEH and 31.4% in EC, respectively (*P* = 0.520). Patients younger than 35 years, those lost more than 10% of their own weight, received combined therapy, or conceived during the follow-up period had a lower probability of recurrence. High recurrence rates were observed in patients with PCOS or obesity.

### Fertility outcomes

After achieving CR, 39 women attempted to conceive. Of these, 28 (71.8%) were referred to ART. In total, 20 (51.3%) patients became pregnant: 11 (28.2%) successfully delivered, 1 (5.1%) was still pregnant, while 8 (20.5%) miscarried (6 were in the first trimester and 2 were in the second trimester) (Table 2).

In the univariate analysis, the pregnancy rate was higher in patients younger than 35 years (58.1% vs. 25.0%, *P* = 0.045). A lower probability of conception was observed in obese patients, those with PCOS, and who lost 10% of their weight. ART and IVF-ET were associated with a high tendency for pregnancy (Table 6).

**Table 6:** Factors related to pregnancy

Factors	Univariate analysis HR (95% CI)	*P*-value	Multivariate analysis HR (95% CI)	*P*-value	Ratio
Age (years): <35 vs. ≥35	3.158 (0.725–13.726)	**0.045**	4.778 (0.671–34.151)	0.114	58.1% vs. 25.0%
AEH vs. EC	0.902 (0.577–1.411)	0.651			46.2% vs. 53.8%
Obesity: No vs. Yes	1.228 (0.504–2.995)	0.651			53.8% vs. 46.2%
Loss-weight:<10% vs. ≥ 10%	0.972 (0.607–1.555)	0.905			50.0% vs. 52.0%
PCOS: No vs. Yes	0.842 (0.265–2.673)	0.770			55.6% vs. 50.0%
Regimen: GnRHa vs. GnRHa combination	0.903 (0.734–1.194)	0.589			40.0% vs. 52.9%
ART: No vs. Yes	0.912 (0.614–1.356)	0.648			45.5% vs. 53.6%
IVF-ET: No vs. Yes	0.877 (0.503–1.531)	0.643			47.1% vs. 54.5%

Notes: PCOS, Polycystic ovary syndrome; AEH, Atypical endometrial hyperplasia; EC, Endometrial carcinoma; GnRHa, Gonadotrophin releasing hormone agonist; ART, assisted reproductive technology; IVF-ET, in vitro fertilization and embryo transfer

### Comparison between EC patients with primary and secondary progestin-resistant

In this study, we categorized the 47 EC patients based on their response to initial progestin treatment over a 6-month period. The classification was as follows: Primary progestin-resistant: Patients who showed SD or PD after 6 months of progestin therapy. Secondary progestin-resistant: Patients who initially showed a PR but did not achieve CR after at least 6-month period progestin therapy following PR. Among the 47 EC patients, 36 were classified as primary progestin-resistant, and 11 were classified as secondary progestin-resistant. We compared the oncological and reproductive out between these two groups (Table 7). The baseline information of these two groups showed no difference (*P* < 0.05). Overall, 91.7% primary progestin-resistant and 90.9% secondary progestin-resistant patients achieved CR with the 6 months (range: 3–12 months) median re-treatment time (*P* = 0.937).

**Table 7:** Comparison between primary and secondary progestin-resistant patients

Characteristics	Primary progestin-resistant (*n* = 36)	Secondary progestin-resistant (*n* = 11)	*P*-value
Complete remission			
Complete remission rate	33 (91.7%)	10 (90.9%)	0.937
Re-treatment time, month (range)	6 (3-12)	6 (3-12)	0.653
Recurrence			
Recurrence rate	10 (30.3%)	4 (40.0%)	0.566
Recurrence time, month (range)	15 (9–48)	24 (12-16)	0.583
Attempts to conceive	25	7	
Live baby delivery	6 (24.0%)	3 (42.6%)	0.327
In pregnancy	1 (4.0%)	0	0.591
Miscarriage	6 (24.0%)	1 (14.3%)	0.583
Total pregnancy rate	6 (52.0%)	4 (57.1%)	0.810

## Discussion

The results of this study suggested that GnRHa-based therapy, used as a fertility-sparing re-treatment method for progestin-resistant EC and AEH patients, achieved a 90.2% response rate. The time from initiation of GnRHa therapy to CR was 6 months (range: 6–12 months), and the recurrence rate was approximately 30% during a median follow-up of 3 years. The pregnancy and live birth rate were about 50% and 30%, respectively, with a median time to pregnancy of 1 years. Adverse effects were minor and there were no recorded deaths due to the disease during the follow-up period.

Progestin carries out its anticancer function by antagonizing estrogen stimulation, inhibiting cell proliferation, promoting differentiation, reversing malignant phenotype [15]. Conservative treatment with high-dose progestin can achieve a good remission rate for young EC patients. However, as the treatment duration extends, progestin receptors decrease, and drug resistance may occur in some patients. For these patients who still wish to preserve their uterus, the question of whether and how to preserve fertility remains a significant challenge.

Alternative drugs that for the fertility-sparing treatment of EC include metformin, LNG-IUD, GnRHa, and AIs, etc [6]. LNG-IUD can provide a local intrauterine progestin concentration that is significantly higher than oral progestin and can exert a therapeutic effect through local action on the endometrium [16]. GnRHa can indirectly affect endometrial cell proliferation through the hormone axis, as well as directly act on GnRH receptors. Hence, GnRHa has an anti-proliferation effect on endometrial cells and can be used to treat endometrial diseases [17]. Letrozole is the third-generation AIs, can reduce estrogen levels by inhibiting its synthesis, leading to a reduction in the growth stimulated in estrogen receptor-positive tumors such as EC [11]. Some studies have attempted to use GnRHa in combination with other methods for conservative treatment of EC, and the effect appears comparable to that of oral progestin, particularly for obese and recurrent patients [7, 18, 19]. In this study, we reported on 61 progestin-resistant patients with EC and AEH treated with GnRHa-based treatment, and over 90% of these cases achieved CR, further validating the feasibility of GnRHa therapy for EC and expanding the range of GnRHa and fertility-sparing treatment’s applications.

In our study, all patients initially received oral progestin therapy, which was subsequently switched to GnRHa treatment. The CR rate reached 90.2%, with 6 months of median time, indicating that most cases could be cured after 6 months of treatment, following two hysteroscopic evaluation, thereby suggesting a rapid and high response rate to the treatment. Especially for AEH patients, CR rate reached nearly 80% after the second course of treatment, and fewer than 30% of patients needed the third hysteroscopic evaluation. This implies less time and cost, fewer side effects, and less endometrial damage, making GnRHa-based therapy an option worth trying and expecting. However, all patients had been treated with progestin beforehand, some patients may have experienced partial reversal and the high CR rate might owing to the combination of the two drugs. It is worth discussing whether GnRHa therapy reached CR based on the previous progestin treatment.

Patients with PCOS and obesity exhibited a lower CR rate in our research, while those with weight loss exceeding 10% showed a higher CR rate, aligning with previous studies [9, 20]. Obesity and PCOS are high-risk factors for EC, often associated with abnormal liver function, abnormal glucose and lipid metabolism, high risk of thromboembolism, and negative influences on pregnancy outcomes [21, 22]. However, GnRHa treatments have minimal impact on body weight and lack side effects like liver damage and thrombosis. Therefore, for overweight, obesity and abnormal metabolic syndrome patients, GnRHa therapy should be given priority to avoid side effects of progestin therapy, promoting weight loss, endometrial remission, and improved pregnancy success rates. While our research found only mild menopausal symptoms as side effects, long-term consequences of GnRHa usage warrant consideration. Questions remain regarding how long the drug should be discontinued, whether and when hormone replacement should be added, whether bone density monitoring should be implemented, and whether calcium and bisphosphate supplements should be added. Accumulating more case experiences will be necessary to answer these queries.

Previous studies have reported recurrence rates ranging from 10–88% [10, 23, 24]. In this study, 40% of AEH patients and 31.4% of EC patients experienced recurrence, with a median recurrence time of 23 months. The recurrence rate was found to be lower in AEH patients, contradicts previous research results. This discrepancy may be due to the small sample size of AEH patients in our study. It is plausible that an expansion of the sample size could yield different results. Recurrence took place 6–77 months after CR, as confirmed by endometrial pathology, with recurrence rate no higher than those of progestin therapy. However, some patients experienced recurrences as early as 6–7 months after achieving CR. Another study has reported recurrence occurring as early as 3–4 months after CR, mandating the need for early follow-up [25]. The latest recurrence in our cohort occurred at 7 years, with other reports indicating as along as 13 years [26, 27]. This high rate of late recurrence necessitates long-term and regular follow-up. Furthermore, hormonal maintenance therapy is crucial for CR patients who do not wish to conceive immediately after completion of treatment.

Research has explored the correlation between age, maintenance therapy, pregnancy, and recurrence, revealing that patients younger than 35 years, those receiving maintenance therapy, and those achieving successful pregnancy can reduce the recurrence rate [28]. All cases in our study had failed previous progestin treatments, and after persistent education, most of them perused maintenance treatment and attempted to conceive, further emphasizing the necessity of regular and long-term follow-up after treatment. Both maintenance therapy and immediate conception were encouraged to decrease recurrence risk. Patients who lost more than 10% of body weight during treatment demonstrated a lower recurrence rate, while patients with BMI > 28 and PCOS exhibited higher recurrence rates than normal patients. These results indicate the crucial role of weight management in the whole period of tumor management, as it can not only increase the remission rate but also reduce the recurrence rate.

Currently, unified standard for the managing recurrence after fertility preservation therapy is lacking. Immediate standard surgical intervention is recommended once recurrence occurs. However, other researchers proposed that when AEH and EC recurred without lesions progression beyond the fertility preservation standard, conservative treatment could still be selected again [10]. In our study, some patients experiencing recurrence were treated with the conservative treatment again after evaluation, and the CR rate was still about 70%. One patient experienced a second recurrence and received the third-time fertility-sparing therapy, also achieved CR, which indicating that fertility preservation remains possible. However, repeated treatments could lead to increased side effects, higher treatment failure probability, poor endometrial receptivity, and lower subsequent pregnancy rate even if the endometrial lesions reversed, which is a challenge we must confront. Therefore, a patient-centric approach considering factors like age, ovarian function, and financial circumstances is supposed to consider before re-treatment.

The pregnancy rate was about 50%, but the live birth rate was suboptimal, with approximately 20% of patients experiencing miscarriage. The pregnancy rate of patients receiving ART exceeded that of natural pregnancies, consistent with previous studies [29, 30]. The relatively low pregnancy rate may stem from the fact that all cases were treated with GnRHa after progestin therapy, and the overall treatment time was over 12 months. Repeated hysteroscopic evaluation and curettage operation may cause endometrial injury or adhesion [31]. Also, it remains unknown whether GnRHa induces irreversible endometrial atrophy. Thus, during conservative treatment, endometrial protection and monitoring should be strengthened, the treatment time should be minimized, and hysteroscopic localization biopsies should be performed to reduce the number and area of uterine operation, thereby minimizing endometrial damage. Other studies have shown that patients with successful pregnancy have a lower recurrence risk, potentially due to the protective effect of long-term high progestin exposure on the endometrium during pregnancy [32]. Hence, once CR is achieved, patients are encouraged to conceive as soon as possible, resorting to ART if necessary.

### Limitation

This study has several limitations. First, the sample size was relatively small, which may affect the generalizability of our findings. Second, the study design was observational, limiting our ability to establish causality. Third, variations in treatment protocols and patient adherence could affect the outcomes, and these factors were not fully controlled in this study. Future research with larger, multi-institutional, randomized controlled trials is necessary to confirm our results.

## Conclusion

In conclusion, with careful evaluation and close monitoring, GnRHa-based fertility-sparing re-treatment can result in a great CR rate and subsequent successful pregnancy and live birth, offering a reliable alternative treatment for EC and AEH patients with progestin-resistance. This provides a new treatment prospect and approach for conservative treatment of EC.

## Data Availability

No datasets were generated or analysed during the current study.
